# The Renin–Angiotensin–Aldosterone System (RAAS): Beyond Cardiovascular Regulation

**DOI:** 10.3390/vetsci12080777

**Published:** 2025-08-20

**Authors:** Agnese Valentini, Romy M. Heilmann, Anna Kühne, Lucia Biagini, Danilo De Bellis, Giacomo Rossi

**Affiliations:** 1 School of Biosciences and Veterinary Medicine, University of Camerino, Via Circonvallazione, 93, 62024 Matelica, Italy; agnese.valentini@unicam.it (A.V.); danilo.debellis@unicam.it (D.D.B.); giacomo.rossi@unicam.it (G.R.); 2 Department for Small Animals, Veterinary Teaching Hospital, College of Veterinary Medicine, University of Leipzig, An den Tierkliniken 23, DE-04103 Leipzig, Germany; romy.heilmann@kleintierklinik.uni-leipzig.de (R.M.H.); anna.kuehne.2000@web.de (A.K.)

**Keywords:** the renin–angiotensin–aldosterone system (RAAS), angiotensin-converting enzyme system (ACE system), angiotensinogen, inflammation

## Abstract

The renin–angiotensin–aldosterone system (RAAS) plays a pivotal role in regulating cardiovascular function, fluid balance, and blood pressure. Recent research has revealed the RAAS’s influence extends beyond cardiovascular physiology, encompassing key roles in inflammation, fibrosis, immune regulation, cancer progression, and organ-specific disease mechanisms. This review provides a comprehensive overview of classical and alternative RAAS pathways, focusing on the dual roles of angiotensin II (Ang II) and angiotensin-(1–7) (Ang 1–7), mediated through AT1R, AT2R, MasR, and MrgD receptors. We discuss molecular signaling cascades, including mitochondrial, nuclear, and caveolae-mediated mechanisms, and explore the impact of RAAS modulation on hepatic fibrosis, vascular remodeling, and autoimmune inflammation. Genetic models and emerging pharmacologic strategies illustrate tissue-specific RAAS actions, emphasizing the therapeutic potential of enhancing the ACE2/Ang 1–7/Mas axis while inhibiting the deleterious ACE/Ang II/AT1R signaling. Furthermore, we highlight implications for veterinary medicine, particularly in canine chronic inflammatory enteropathies, where RAAS dysfunction may contribute to treatment resistance. Understanding RAAS complexity and inter-receptor crosstalk is essential for developing new therapeutic strategies targeting cardiovascular, hepatic, and inflammatory diseases in both human and veterinary contexts.

## Simple Summary

The renin–angiotensin–aldosterone system (RAAS) helps control blood pressure and fluid balance in animals and humans. But it does much more than that. This system also affects how organs respond to injury and disease. It plays a part in inflammation, fibrosis (tissue scarring), cancer, and immune system responses. This article reviews how RAAS works in the body and how it can be targeted to treat disease. RAAS has two main branches: one promotes harmful effects like high blood pressure and inflammation, while the other helps protect the body by reducing damage and supporting healing. These effects happen through different molecules like angiotensin II and angiotensin-(1–7), which bind to specific cell receptors. In the liver, RAAS affects how fibrosis forms, and in the gut, it may influence conditions like inflammatory bowel disease in dogs. By better understanding how RAAS functions in different tissues, scientists and veterinarians can design new drugs that block the harmful parts of RAAS while enhancing its protective actions. This is important for developing future treatments for heart, liver, and intestinal diseases in both pets and people.

## 1. Introduction

The renin–angiotensin–aldosterone system (RAAS) regulates blood pressure, fluid balance, and cardiovascular health. In addition to its role in circulation, the RAAS also influences cancer biology, affecting cell growth, migration, death, and metastasis, as well as playing a role in inflammation, fibrosis, coagulation, and several disease processes [1,2,3]. The RAAS functions through a cascade of enzymatic events and receptor interactions. Several drugs are available that are based on RAAS interference. These include angiotensin-converting enzyme (ACE) inhibitors and angiotensin receptor blockers (ARBs). Renin is an aspartyl protease primarily secreted by the juxtaglomerular cells of the kidneys, where it catalyzes the conversion of angiotensinogen into angiotensin I (Ang I). In addition to renal synthesis, extrarenal tissues such as the uterus, placenta, testicles, adrenal glands, retina, and submandibular glands have also been shown to produce renin [4]. Renin is released from intracellular vesicles in the juxtaglomerular apparatus in response to specific physiological triggers, including a decrease in systemic blood pressure, reduced blood sodium levels, or activation of the sympathetic nervous system via β1-adrenergic receptors. Once released, renin cleaves circulating angiotensinogen (Ang) into angiotensin I (Ang I). Although renin is part of the non-inhibitory serpin (serine protease inhibitor) superfamily, it functions as an active aspartyl protease rather than an inhibitor [5]. Angiotensinogen is primarily synthesized and secreted by the liver but is also produced in other tissues, including the brain, lungs, kidneys, vasculature, and heart [2].

Angiotensin-converting enzyme (ACE) is a dipeptide carboxypeptidase responsible for converting angiotensin I (Ang I) to angiotensin II (Ang II). In contrast, ACE2 is a monocarboxypeptidase that converts Ang II to angiotensin-(1–7) (Ang 1–7), a peptide with opposing biological effects. Ang II is a potent vasoconstrictor that effectively increases systemic blood pressure and also exerts pro-inflammatory, proliferative, and pro-fibrotic effects. These actions define the ‘classical’ arm of the renin–angiotensin–aldosterone system (RAAS). Alternatively, the ‘non-classical’ or ‘protective’ RAAS pathway is centered on ACE2, which also converts Ang I to Ang 1–9, a precursor of Ang 1–7 [5,6]. Ang 1–7 exerts its effects primarily through binding to the Mas receptor, promoting vasodilation and exerting anti-inflammatory and antifibrotic actions. The balance between the classical ACE/Ang II/AT1R axis and the alternative ACE2/Ang 1–7/Mas axis is critical for maintaining cardiovascular and systemic homeostasis [6,7]. Several peptidases, including thimet oligopeptidase (TOP), neprilysin (NEP), and prolyl endopeptidase (PEP), are capable of converting angiotensin I (Ang I) into angiotensin-(1–7) (Ang 1–7). ACE2 also hydrolyzes Ang II directly to generate Ang 1–7 [6,8]. Additionally, Ang I can be cleaved by ACE2 to form Ang 1–9, which can then be processed by ACE or NEP into Ang 1–7. Angiotensin III (Ang III or Ang 2–8) is produced from Ang II by aminopeptidase A (APA) and retains the ability to bind AT1 receptors (AT1R), inducing similar pressor effects, with a more pronounced impact on the central nervous system [9]. Further cleavage of Ang III results in angiotensin IV (Ang IV or Ang 3–8), which binds to AT4 receptors (AT4R) and promotes vasodilation in cerebral and renal vascular beds, enhancing sodium excretion and renal perfusion [10]. Angiotensin A (Ang A), a bioactive variant of Ang II, can bind both AT1R and AT2R, and is further metabolized by ACE2 into alamandine [11]. Alamandine can also be derived via decarboxylation of Ang 1–7 and exerts biological effects through the Mas-related G protein-coupled receptor D (MrgD) [12]. The Ang A/alamandine/MrgD axis thus represents a novel and emerging component of the expanded RAAS network [13].

## 2. Recent Advances in Renin–Angiotensin System Research

In recent years, the understanding of the renin–angiotensin system has expanded well beyond its classical role in blood pressure regulation and fluid balance, revealing a complex network of peptides and receptors with diverse physiological and pathological roles. In addition to the well-known angiotensin II and its AT1 receptor, newer angiotensin peptides such as Ang III, Ang IV, Ang A, and alamandine signal through additional receptors, including AT2 and Mas. These receptors exert various effects, including vasodilation, anti-inflammatory action, and neuroprotection. This increased complexity of the ligand-receptor relationship indicates that the ACE2/Angiotensin-(1-7)/Mas receptor axis is a pivotal counter-regulatory pathway that balances the harmful ACE/Ang II/AT1 receptor pathway. The axis exerts beneficial vasodilatory, anti-fibrotic, and anti-inflammatory effects, which result in a significant alteration of the therapeutic landscape [14,15].

It is important to note that the RAS functions not only systemically but also locally within tissues. Tissue-specific RAS (tRAS) systems have been identified in organs such as the heart, kidney, brain, adipose tissue, and the immune system. These local axes have been demonstrated to exert influence on organ-specific pathologies, including, but not limited to, cardiac remodeling, renal fibrosis, metabolic syndrome, neurodegenerative disease, and immune-mediated inflammation. The brain possesses an autonomous renin–angiotensin system (RAS), which has been demonstrated to possess unique receptor profiles and peptide production. contributing to neurological regulation and disease [15,16,17].

At the molecular level, receptor interactions—including crosstalk between the AT2 receptor and Mas receptor—and subcellular receptor localization in mitochondria and nuclei add layers of regulatory control. It is evident that epigenetic mechanisms, including DNA methylation and histone modifications, dynamically regulate critical RAS components, such as ACE and ACE2. This regulatory process contributes to interindividual variability and provides novel therapeutic opportunities through the utilization of epigenetic modulators [15,16,18].

The field of clinical translation has evolved in parallel with these mechanistic insights. Conventional pharmaceuticals such as angiotensin-converting enzyme (ACE) inhibitors and angiotensin receptor blockers (ARBs) remain fundamental, yet novel therapeutic interventions targeting AT2 and Mas receptors, recombinant human ACE2, and epigenetic drugs are in development. The focus is shifting towards the utilization of tissue-targeted delivery and personalized medicine, with the objective of enhancing efficacy and reducing systemic adverse effects [14,15,19].

Finally, significant species-specific differences in the RAS—such as renin enzyme specificity, the presence of receptor isoforms, and alternative Ang II production pathways like chymase—pose challenges for preclinical research and drug development, underscoring the need for careful translation from animal models to humans [14,15].

The contemporary perspective posits the RAS as a complex, context-dependent system, characterised by its intricate regulation at molecular, cellular, tissue, and epigenetic levels. This sophisticated regulatory network facilitates the development of innovative and precise therapeutic strategies, offering significant potential for the treatment of cardiovascular, renal, metabolic, inflammatory, and neurological diseases.

## 3. Species Differences in RAAS and Their Physiological and Pathological Impacts

The renin–angiotensin–aldosterone system (RAAS) exhibits substantial variations in enzymatic composition, molecular pathways and receptor subtypes across different species, thereby influencing its physiological and pathological functions. It is important to note that renin demonstrates considerable structural variability across different species. This necessitates the development of species-specific inhibitors [15]. While angiotensin peptides are well conserved in mammals, more pronounced divergence is evident in lower vertebrates [15,20]. The angiotensin-converting enzyme (ACE) pathway is characterised by significant conservation, yet there is considerable heterogeneity amongst tissue-specific non-ACE pathways. For instance, chymase-mediated Ang II formation exhibits marked differences amongst species; chymase is highly active in humans and dogs but inactive or Ang II-degradative in rodents [8,15,21,22]. AT1 receptors also exhibit interspecies differences: humans express a single AT1 receptor, whereas rodents express two (AT1A and AT1B), which affects tissue distribution and gene regulation [15,23,24,25,26,27]. These molecular and anatomical distinctions affect the way in which the RAAS regulates blood pressure, renal function, and vascular tone, ultimately influencing disease manifestations and the efficacy of pharmacology. For example, ACE inhibitors are less effective in species with significant chymase-mediated Ang II generation [8,28,29]. Recognizing these differences is essential for interpreting animal models, guiding species-appropriate drug development, and enabling precision therapies that exploit tissue- and species-specific RAAS dynamics for broader clinical applications, including organ fibrosis and immune-mediated diseases [8,29]. Having established these fundamental inter-species differences, it becomes essential to examine how the balance between different RAAS axes determines physiological and pathological outcomes at the tissue level.

## 4. Functional Axes and Balance

Research indicates that the function of various organs and organ systems may depend on a balance between the activation of the ACE/Ang II/AT1R pathway and the ACE2/Ang 1–7/Mas receptor pathways. Imbalances favoring the ACE/Ang II/AT1R pathway can result in cardiovascular diseases. However, the function of this axis extends well beyond cardio-renal and vascular mechanisms due to its pleiotropic effects, which can impact a wide range of diseases [30,31,32]. The activation of the ACE/Ang II/AT1R pathway is associated with inflammation, oxidative stress, fibrosis, and hypertrophy [27].

Ang 1–7, an active heptapeptide, is another Ang derivative [33] that can interact with the endogenous Mas receptor (MASR). The activation of this receptor can counteract the actions of Ang II and offset most of its undesirable effects. Therefore, MASR has positive cardiovascular effects and can decrease systemic blood pressure. In addition to its initially identified cardiovascular and renal functions, Ang 1–7 exhibits various functions in different tissues and organs [34].

## 5. Localization and Binding Specificity of AT2 and Mas Receptors

The Angiotensin II Type 2 Receptor (AT2R) and the Mas receptor represent critical components of the protective arm of the renin–angiotensin–aldosterone system (RAAS), counterbalancing the classical Angiotensin II Type 1 Receptor (AT1R) axis. AT2R is expressed in a variety of regions and organs. Its expression is particularly prevalent in the central nervous system, including brain regions such as the locus coeruleus, amygdala, and brainstem nuclei. In addition, AT2R is also expressed in peripheral organs such as the heart, kidney, vasculature, and adrenal glands. Its expression is dynamic, increasing during physiological adaptations (e.g., pregnancy) and pathological states (e.g., hypertension or cardiac injury), highlighting a potential protective role [35,36].

The Mas receptor also exhibits broad tissue distribution, with unique subcellular localization to mitochondria and nuclear envelopes in neural and glial cells, implying specialized intracellular signaling functions. It co-localizes frequently with AT2R in renal proximal tubules, regulating nitric oxide (NO) signaling and sodium homeostasis [27]. Angiotensin II binds AT2R with specificity influenced by receptor structure, redox status, dimerization (including heterodimers with AT1R and bradykinin B2 receptors), and expression level modulation, all contributing to its vasodilatory and anti-inflammatory effects [25,37]. Similarly, Angiotensin-(1-7) binds the Mas receptor with high affinity, its effects enhanced through receptor heteromerization (Mas–AT1R and Mas–AT2R complexes) and distinct intracellular trafficking pathways that affect downstream signaling [27].

Recent advances underscore the synergistic actions of AT2R and Mas receptors in cardiovascular and renal protection through anti-inflammatory, anti-fibrotic, and anti-proliferative mechanisms. Their coordinated modulation represents a promising target for novel therapeutics in cardiovascular and metabolic diseases, with pharmacological agents such as ACE inhibitors and ARBs augmenting this protective axis [38]. Studies have shown that the Mas antagonist, A779, can block most of the actions of Ang 1–7 [34,39,40,41]. Furthermore, the effects of Ang 1–7 are not found in Mas-deficient animals [39,42,43,44]. MrgD can also mediate some of the actions of Ang 1–7 [39,41,42,43]. Therefore, the Ang 1–7/Mas receptor interaction can regulate several signaling pathways, including the ERK and phosphoinositide 3-kinase (PI3K)/protein kinase B (AKT) pathways. It can also affect downstream effectors, including NO, cyclo-oxygenase-2 (COX-2), and forkhead box O1 (FOXO1). These mechanisms allow Ang 1–7 to act on improving pathological disorders such as inflammation and fibrosis in various organs such as the liver, lungs, and kidneys. Due to its potential to prevent angiogenesis and cell proliferation, Ang 1–7 can be considered a potential anticancer effector [11].

## 6. Selective Regulation of ACE/Ang II/AT1R and ACE2/Ang 1–7/Mas Receptor Pathways

### 6.1. ACE/Angiotensin II/AT1 Receptor Pathway (Classical Axis)

This pathway promotes vasoconstriction, inflammation, fibrosis, and hypertrophy. It is regulated via transcriptional mechanisms, such as pro-inflammatory cytokines like TNF-α and IL-1β, enhancing AT1R expression through NF-κB. Post-translational modifications affect receptor sensitivity, and AngII-mediated positive feedback increases AT1R levels while downregulating ACE2 via AT1-ERK/p38 MAPK signaling, reinforcing harmful effects. Local tissue-specific AngII synthesis by enzymes such as chymase adds to pathological effects independently of systemic ACE activity [43].

### 6.2. ACE2/Angiotensin-(1-7)/Mas Receptor and AT2 Receptor Pathway (Protective Axis)

ACE2 converts AngII into Ang-(1-7), shifting the balance toward vasodilation, anti-inflammatory, and anti-fibrotic actions primarily through nitric oxide signaling by Mas receptor activation. AT2R activation contributes to anti-inflammatory and anti-proliferative effects and is upregulated under stress conditions. Pharmacological agents like ACE inhibitors (ACEi), AngII receptor blockers (ARBs), mineralocorticoid receptor antagonists, and emerging ACE2 activators or MasR and AT2R agonists promote this protective arm [45].

### 6.3. Mechanisms Maintaining Balance

Balance is maintained through substrate competition between ACE and ACE2, receptor crosstalk with homo- and heterodimerization among AT1R, AT2R, and MasR, and tissue-specific expression patterns. Pathological conditions induce dynamic shifts in receptor expression, often elevating protective receptors as compensatory responses [43].

### 6.4. Genetic and Epigenetic Regulation

Polymorphisms in the ACE and ACE2 genes influence enzyme levels and disease susceptibility. Epigenetic modifications, including DNA methylation and histone marks, regulate expression of RAS components and can be modulated by environmental and lifestyle factors, representing emerging therapeutic targets through “epidrugs” [46].

## 7. Tissue-Specific Roles of Angiotensin II and Local RAS Autonomy

**Figure 1:**
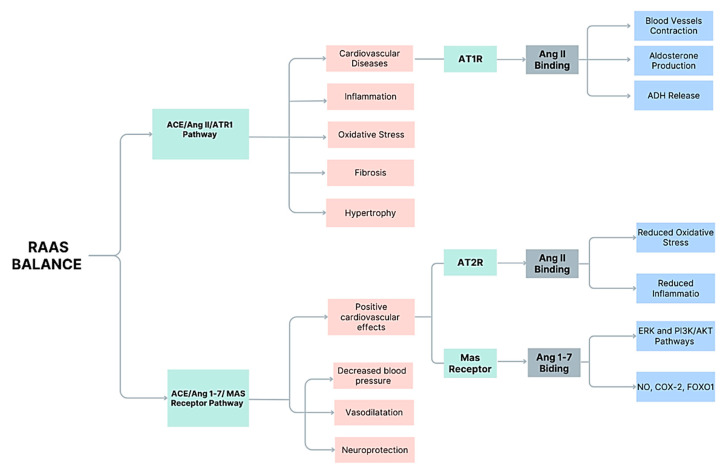
Schematic representation of the renin–angiotensin–aldosterone system (RAAS), highlighting two main counter-regulatory pathways. The ACE/Ang II/AT1R pathway promotes vasoconstriction, aldosterone production, antidiuretic hormone (ADH) release, oxidative stress, fibrosis, hypertrophy, and contributes to cardiovascular diseases. In contrast, the ACE/ Ang-(1-7)/MAS receptor pathway exerts protective effects, including vasodilation, blood pressure reduction, neuroprotection, and overall positive cardiovascular outcomes. Additionally, Ang II binding to AT2 receptors attenuates oxidative stress and inflammation through ERK and PI3K/AKT signaling pathways. The balance between these pathways is essential for cardiovascular homeostasis and disease prevention. (Ang = angiotensinogen; ERK = extracellular signal-regulated kinase; PI3K = phosphoinositide 3-kinase; AKT = protein kinase B; NO = nitric oxide; COX-2 = cyclo-oxygenase-2; FOXO1 = forkhead box O1).

RAS components differ across tissues, with local AngII and Ang-(1-7)production influencing organ-specific functions such as neuroprotection, cardiovascular remodeling, and renal sodium handling. This spatial specificity informs selective drug targeting strategies [43,47]. Angiotensin II (Ang II) exhibits diverse and complex roles beyond systemic blood pressure regulation, functioning as an endocrine, paracrine, and autocrine factor across multiple tissues (Figure 1).

### 7.1. Central Nervous System (CNS)

The brain harbors a local renin–angiotensin system (RAS), independent of circulating Ang II, with receptors AT1R, AT2R, MasR, and AT4R mediating opposing actions. Ang II influences cardiovascular control, fluid balance, cognition, and neuroprotection. AT1R promotes oxidative stress and inflammation, contributing to neurodegeneration, whereas AT2R and AT4R support neuroprotective and cognitive functions [48].

### 7.2. Immune System

Ang II drives immune cell activation, differentiation, and recruitment by acting via angiotensin receptors on T cells, macrophages, dendritic cells, and NK cells. It induces pro-inflammatory cytokines (IL-6, TNF-α), oxidative stress, and NF-κB signaling, exacerbating vascular inflammation and autoimmune diseases. Ang II also enhances innate immune memory and T cell autocrine signaling, perpetuating inflammation [48].

### 7.3. Metabolic Tissues

In adipose tissue, Ang II promotes inflammation, adiposity, insulin resistance, and dysregulated lipid metabolism. In the pancreas, it reduces blood flow and insulin secretion, potentially triggering diabetes. In skeletal muscle, Ang II impairs insulin-stimulated glucose uptake and blood flow, worsening insulin sensitivity, effects counteracted by Angiotensin-(1-7) [49,50].

### 7.4. Cardiovascular System

Ang II via AT1R stimulates cardiac hypertrophy, fibrosis, and vascular smooth muscle proliferation. Conversely, AT2R antagonizes these effects, providing antifibrotic, anti-inflammatory, and antioxidative actions. Ang II increases vascular permeability and adhesion molecule expression, linking hypertension to vascular injury and inflammation [51].

### 7.5. Kidney

The renal RAS is highly responsive to Ang II, which promotes sodium retention, fibrosis, oxidative stress, and inflammation, contributing to chronic kidney disease progression. AT2R and Mas receptors co-localize in proximal tubules to regulate sodium excretion and nitric oxide signaling, modulating renal function and blood pressure [19,52,53].

Overall, Ang II acts as a key integrative hormone and cytokine, with tissue-specific receptor expression patterns and signaling pathways dictating its diverse roles in physiology and pathology. This points to the critical importance of context-dependent modulation of Ang II signaling in targeted therapeutics. The theoretical understanding of these balanced mechanisms is directly applicable to clinical practice, as evidenced by the therapeutic efficacy of ACE/Ang II/AT1R system inhibitors, as demonstrated by decades of clinical evidence.

## 8. Clinical Evidence and Benefits of AT1R/Ang II Inhibitors

Inhibiting the Angiotensin II Type 1 Receptor (AT1R) or blocking Angiotensin II (AngII) binding in the renin–angiotensin–aldosterone system (RAAS) leads to significant symptom relief and clinical improvements across several diseases [54]. Angiotensin II Receptor Blockers (ARBs), such as losartan and olmesartan, act by counteracting the harmful ACE/AngII/AT1R axis, reducing vasoconstriction, inflammation, fibrosis, and hypertrophy, while enhancing protective pathways mediated by AT2 and Mas receptors [55,56]. This shift improves vascular function and reduces oxidative stress, translating clinically into better blood pressure control, fewer side effects, relief of heart failure symptoms, and renal protection in diabetic nephropathy by stabilizing kidney function and lowering proteinuria [36,55,57,58,59]. Emerging evidence also suggests RAAS modulation may alleviate chronic neuropathic pain by targeting AT2 receptors [55,59,60,61]. These effects collectively improve patients’ quality of life by restoring molecular and functional balance beyond traditional physiological measures [60].

## 9. AT1R and AT2R: Opposing Effects

Within the RAAS, the two predominant receptors, AT1R and AT2R, have opposing actions. They are G protein-coupled receptors that have equivalent affinities for Ang II but exhibit markedly different actions within the body [62,63]. AT1R has long been known to mediate Ang II effects, including inflammation, cell proliferation, oxidative stress, and sympathetic activation, contributing to systemic hypertension and organ fibrosis. AT2R, discovered more recently, plays a more protective role [64,65]. Though still an area of intensive research, AT2R plays a protective role within RAAS, by counteracting AT1R, reducing oxidative stress and inflammation, while promoting vasodilation and neuroprotection. Its benefits make it a promising drug target for various diseases [25].

Another significant RAAS receptor is MasR, which, similar to AT2R, has protective effects. MasR interacts with Ang 1–7, decreases systemic blood pressure, fibrosis, and sympathetic tone, but increases vasodilation and nitric oxide production. Paradoxically, there is also a variant of MasR called MrgD that does not interact with Ang 1–7 but instead with alamandine, a compound that shares the same protective characteristics [33,66].

Ang II has very powerful effects by binding to ATR1: (i) it stimulates the contraction of blood vessels, raising systemic blood pressure; (ii) it stimulates the adrenal glands to produce aldosterone, which causes the kidneys to retain more sodium and water, further increasing systemic blood pressure; and (iii) it promotes the release of antidiuretic hormone (ADH), which affects water reabsorption in the kidneys. All these mechanisms aid in maintaining a stable balance in systemic blood pressure and fluid volume [3,67,68].

In addition to its main function, Ang II plays a key role in many cardiovascular diseases by influencing cell growth and vascular remodeling [69,70]. It binds to the AT1R receptor, triggering a series of responses that can promote systemic hypertension and organ damage. At the cellular level, Ang II can cause oxidative stress by increasing the production of reactive oxygen species (ROS), which damage cells and promote inflammation. Ang II can also affect mitochondria, the energy centers of cells, causing their fragmentation and reducing energy production, with negative effects on vascular smooth muscle cell function [71,72,73].

The major functions of Ang II in the cardiovascular system are mediated by the type 1 receptor (AT1R), the activation of which triggers a complex network of intracellular signaling. This process contributes to the development of systemic hypertension, cardiovascular remodeling, and organ damage. The binding between Ang II and AT1R involves the heterotrimeric G-proteins Gq/11, G12/13, and Gi and involves second messengers such as inositol triphosphate, diacylglycerol, arachidonic acid, and ROS [72,74] This interaction leads to the activation of phospholipases C, A, and D, followed by the phosphorylation of tyrosine kinases and the stimulation of mitogen-activated protein kinase (p42\/p44 MAPK)\/extracellular signal-regulated kinase (ERK1\/2) [75]. These molecular events have a direct impact on the growth of cardiomyocytes, fibroblasts, and vascular smooth muscle cells (VSMCs) and actively contribute to cardiovascular remodeling processes [76].

In recent years, scientific evidence has shown that Ang II plays a key role in the activation of new cellular signaling pathways [77,78]. Wnt/β-catenin pathway: Ang II, through the AT1R, induces the expression of the growth factor WISP1 (WNT1 Inducible Signaling Pathway Protein 1), which activates a signaling cascade involving CREB (cAMP response element-binding protein) and the NOX2\/Akt\/GSK-3β\/β-catenin\/TCF\/LEF pathway in cardiomyocytes. This process promotes cardiac hypertrophy and contributes to cardiac remodeling mechanisms [58,79]. Notch signalling pathway: AT1R-mediated activation of the Notch pathway has been confirmed in HEK293 cells and human VSMCs. AT1R stimulation promotes the activation of NADPH oxidase (NOX), which increases oxidative stress and modulates Notch signaling activity. This signaling pathway is involved in the regulation of gene transcription and, once activated, promotes the translocation of the intracellular Notch domain (NICD) into the nucleus where it modulates the expression of genes associated with vascular remodeling and migration of VSMCs [78,80]. This contributes to neo-intima formation in the processes of atherosclerosis and vascular restenosis. It regulates VSMC phenotype balance, impacting vessel stability and promoting hypertrophy and fibrosis. These mechanisms are key to understanding cardiovascular disease and may lead to new antihypertension and other vascular treatment options [77,78]. To fully understand how these antagonistic receptors exert their opposing effects, it is necessary to examine the intracellular molecular mechanisms that mediate Ang II signaling.

## 10. Intracellular and Mitochondrial Effects of Ang II

Ang II regulates intracellular communication, especially in mitochondria, where it boosts ROS production. These ROS enhance Ang II signaling, driving inflammation and hypertrophy. Mitochondrial dysfunction also dysregulates ATP production, impairing VSMC metabolism, which has a key role in cardiovascular disease and vascular remodeling [76]. Ang II influences mitochondrial fission, dividing mitochondria to support energy balance, cell division, and metabolic adaptation. This process aids stress response and mitophagy, but excessive fission is linked to neurodegenerative and cardiovascular diseases [81]. At the mitochondrial level, Ang II stimulates the phosphorylation of dynamin-related protein 1 (Drp1), which promotes mitochondrial fission. When excessive, this process compromises mitochondrial function, increasing cellular susceptibility to oxidative stress and promoting VSMC proliferation and migration, with direct effects on vascular remodeling and hypertrophy [82].

## 11. Caveolae and AT1R Signaling

Ang II is also closely associated with caveolae, cholesterol- and caveolin-rich plasma membrane micro-domains that function as highly organized signaling platforms. These compartments regulate intracellular communication by facilitating AT1R trafficking and signaling, thereby influencing the duration and intensity of its activation. In VSMCs and endotheliocytes, caveolae contribute to the regulation of vasoconstriction and vascular remodeling, creating a highly dynamic environment for Ang II-induced signaling [83,84,85,86].

## 12. Nuclear Effects and Fibrosis

The opposing actions between the “classic” and “alternative” RAAS pathways (agonists/antagonists) are the basis of maintaining a balance (homeostasis). Disruption of “alternative” RAAS pathways can have several negative effects on different organ systems and is often accompanied by fibrosis and loss of organ function.

In addition to the mechanisms described above, Ang II acts at the nuclear level by activating the nuclear AT1R, which regulates cardiac fibroblast proliferation and collagen synthesis, thereby promoting cardiac fibrosis. In addition, Ang II modulates histone deacetylase (HDAC) activity, affecting gene expression and contributing to cardiac and vascular hypertrophy [87,88].

Another key mechanism regulated by Ang II is S-nitrosylation, a post-translational modification that affects the function of several proteins, including soluble guanylate cyclase (sGC), reducing cGMP production in VSMCs and enhancing the pro-hypertensive effects of Ang II. The AT1R is also subject to S-nitrosylation, which directly affects its signaling activity [84,88,89].

The development of transcriptomic and proteomic technologies has made it possible to identify specific Ang II-regulated genes and proteins and to associate them with the pathological phenotypes induced by this molecule. These studies have led to the discovery of new mediators of Ang II signaling in different tissues, improving our understanding of its impact on cardiovascular disease [90,91]. These complex molecular mechanisms manifest differently across various tissues, as elegantly demonstrated by studies using tissue-specific knockout models that have revealed cell-specific roles of RAAS.

## 13. Tissue-Specific Roles and Gene Knockouts

Finally, genetic engineering using tissue-specific AT1R knockout mouse models (AT1R-/-) has allowed detailed investigation of Ang II signaling in different cell types. These studies have revealed significant differences in the signaling mechanisms between different cells, highlighting their contribution to many pathologies. For example, AT1R signaling in adventitial fibroblasts, but not in VSMCs or endothelial cells, was shown to be the major factor responsible for Ang II-induced media thickening and hyperplasia in the ascending aorta [92,93].

## 14. Role of RAAS in Hepatic Fibrosis Development

Liver fibrosis can result from chronic liver diseases (e.g., chronic active inflammation) that cause excessive deposition of extracellular matrix and formation of regenerative nodules. Compression of the blood vessels results in structural changes, vascular remodeling, and loss of liver function. Hepatic stellate cells (HSCs) and hepatic sinusoidal endothelial cells (LSECs) can react to vascular resistance and portal venous pressure changes. Activated HSCs release excess collagen, proteoglycan, and ECM components [94], which accumulate in the Dissé space. Contraction of HSCs increases hepatic sinusoid pressure, leading to portal hypertension, liver fibrosis, and cirrhosis [95]. Current treatment options to prevent or halt the progression of liver fibrosis are largely ineffective, and modulation of HSC activity and autophagy might be a novel target. ACE2 overexpression after injecting a liver-specific, recombinant adenovirus-associated ACE2 vector (rAAV2/8-ACE2) is associated with downregulation of platelet-derived growth factor (PDGF), endothelial growth factors, angiopoietin-2, and Ang II, all of which promote collagen formation, inflammation, vascular remodeling, and fibrogenesis. Induction of anti-inflammatory interleukin (IL)-10 and apoptosis of ECM-producing HSCs reduces collagen formation and slows fibrosis, which is supported by the anti-inflammatory, antioxidant, and antifibrotic actions of increasing Ang 1–7 levels. ACE2-mediated HSC autophagy can be regulated via the AMPK/mTOR signaling pathway, as shown in a murine model of hepatic fibrosis and sinusoid remodeling72. Overexpression of ACE2, inhibiting HSC activity and promoting HSC apoptosis, attenuates hepatic fibrosis. Injection of rAAV2/8-ACE2 also inhibits the expression of VEGF, angiopoietin-2, and PDGF, which are angiogenic factors that stimulate hepatic fibrosis. Inflammatory cells, fibrotic changes, and collagen deposition were also lower in rAAV2/8-ACE2-treated mice. Low ACE2 activity can worsen hepatic fibrosis [96]), and administration of recombinant ACE2 supports improvement [95]. Thus, ACE2 is central in attenuating intrahepatic angiogenesis and intrahepatic sinusoidal resistance, and RAAS pathways could be pharmacologic targets for hepatic fibrosis [97]. Carvedilol, a β-blocker used to treat systemic hypertension, alleviates fibrosis development by inhibiting Ang II-induced HSC proliferation and activation. Modulation of other RAAS components (inhibition of ACE/Ang II/AT1R and stimulation of ACE2/Ang 1–7/Mas signaling pathways) was also shown to reduce hepatic fibrosis in a rodent model of hepatic fibrosis.

Further progress is expected over the next decade from research into the pharmacologic modulation of “traditional” and “alternative” RAAS. Currently, none of the RAAS-interfering drugs available are licensed for the treatment of hepatic fibrosis, but promising approaches and future therapeutic options are on the horizon.

The role of RAAS in hepatic fibrosis represents just one example of its influence on systemic inflammatory processes. Indeed, inflammation modulation constitutes one of the most pervasive mechanisms through which RAAS influences the pathogenesis of numerous diseases.

## 15. Inflammation, Immunomodulation, and Related Disorders

Ang II-mediated intracellular communication occurs through distinct but interrelated mechanisms that are critical to the processes of cellular adaptation and response in cardiovascular disease. Although less extensively studied than AT1R, the AT2R is recognized for its function in counteracting and modulating AT1R-mediated signaling. Its major actions include vasodilation (through stimulation of NO and cGMP production), natriuresis, anti-angiogenesis, anti-proliferative effects, and reduction of fibrosis in various tissues [37,98,99,100].

Recent studies suggest that AT2R activation occurs independently of traditional G-protein and β-arrestin-mediated signaling pathways [35]. Ang II binding to AT2R activates several phosphatases, including MAPK phosphatase-1 (MKP-1) and SHP-1, which inhibit proliferative and pro-inflammatory pathways, contributing to the anti-proliferative and pro-differentiative effects of the receptor [101,102]. Another key mechanism is the potentiation of bradykinin signaling, a vasoactive peptide involved in cardiovascular protection and reduction of inflammation. Finally, an important aspect of “alternative” RAAS signaling is the neuroprotective role of the AT2R, which has been implicated in neural regeneration and defense against oxidative stress, underlining its importance not only at the cardiovascular level but also within the nervous system and repair of oxidative stress damage [81] RAAS maintains a balance between Ang II and Ang 1–7 for cardiovascular protection. ACE inhibitors and sartans lower Ang II while enhancing the beneficial effects of Ang 1–7. Beyond systemic blood pressure regulation, RAAS plays a role in inflammation, cancer progression, and disease mechanisms. The ACE/Ang II/AT1R axis drives pro-inflammatory processes, angiogenesis, and fibrosis, contributing to cardiovascular and oncological disease progression. AT1R activation also stimulates NAD(P)H oxidase, increasing ROS and sustaining inflammation. Ang II-mediated signaling in vascular, cardiac, renal, and cerebral systems, along with inflammation, relies on intricate RAAS interactions. Understanding these mechanisms is key to developing therapies for cardiovascular and inflammatory diseases.

Ang II-driven inflammation is crucial in intracranial, aortic-abdominal (AAA), and thoracic (TAA) aneurysm formation and rupture [103]. Ang II infusion is known to increase the LysM+ monocyte population, promote iNOS expression, and cause eNOS uncoupling, leading to increased endothelial nitro-oxidative stress [104]. Ang II promotes hematopoietic stem cell and immune cell activation, influencing leukocytes, microbiota, and immune responses in intestinal inflammation. It also drives immune signaling in vascular inflammation, impacting hypertension, cardiac hypertrophy, and other organs. Regulatory T lymphocytes play an essential role in preventing Ang II-induced hypertension and vascular damage by modulating systemic inflammation and contributing to vascular stability [105,106].

Increased Ang II activity ensures an effective immune response to pulmonary infections, and hypoxia-induced and Ang II-mediated vasoconstriction prevents the shunting of blood with pneumonia or lung injuries. RAAS also plays a central role in maintaining the oxygen supply and preventing organ failure with acute respiratory distress syndrome (ARDS), a pulmonary overreaction to toxic or other stimuli due to pro-inflammatory and cytokine-mediated tissue damage. Patients with increased ACE activity were shown to have a higher risk of ARDS, whereas overexpression of ACE2 has a protective effect, and ACE2-/- mice have a more severe course of ARDS that responds to recombinant ACE2 administration. However, ACE2 is also a possible entrance mechanism for SARS-CoV-2 [31].

The protective arm of RAAS, mediated by the ACE2/Ang 1–7/Mas axis, counteracts the pro-inflammatory effects of Ang II, promoting vasodilation and cardiovascular protection. AT2R plays a key role by exerting anti-proliferative, anti-inflammatory, and antioxidant effects, reducing oxidative stress. Its activation lowers NAD(P)H oxidase activity, ROS formation, and inflammatory cytokine synthesis, mitigating inflammation-related damage. Alamandine/MrgD forms an additional RAAS pathway that counteracts the harmful effects of Ang II. RAAS maintains a dynamic balance between pro-inflammatory (ACE/Ang II/AT1R) and anti-inflammatory (ACE2/Ang 1–7/Mas, AT2R) RAAS pathways. Understanding this equilibrium is vital for health and exploration of novel therapies for RAAS-related diseases [10].

RAAS inhibitors, traditionally used to treat cardiovascular disease, may offer benefits in cancer therapy by modulating cancer-associated inflammation [107,108,109,110]. In addition, their use may prove useful in the treatment of autoimmune diseases, highlighting the role of the RAAS in the control of immune and inflammatory processes [111,112].

Similar to inflammatory bowel diseases (IBD) in humans, canine chronic inflammatory enteropathies (CIE) can cause severe maldigestion, malabsorption, and intestinal water loss through diarrhea and/or vomiting. These can significantly affect the patient’s quality of life. Water homeostasis is crucially dependent on RAAS-mediated intestinal and renal electrolyte and water absorption, and the colon can compensate for large losses of water from the proximal small intestine. However, counter-regulation of RAAS-activated colonic electrolyte transport capacities is ineffective in both human IBD [113]) and canine CIE [114] despite systemic RAAS upregulation [115,116], possibly due to “RAAS resistance”. Targeting RAAS-dependent colonic electrolyte transport might thus be a future avenue for adjunctive therapy in human IBD, canine CIE, and potentially other causes of intestinal inflammation.

## 16. RAAS and Cancer

Scientific evidence has also implied that RAAS is involved in the development and progression of cancer. For example, activation of the AT1R may promote MAPK and STAT3 signaling in prostate cancer, contributing to tumor proliferation [117]. In ovarian cancer, increased AT1R expression is associated with tumor angiogenesis, suggesting a possible role in tumor expansion and aggressiveness [118,119].

## 17. Conclusions and Perspectives

Ang II signaling is a complex process involving ligands, receptors, intracellular pathways, organelle interactions, and post-translational modifications. These mechanisms vary by cell type and tissue, making RAAS understanding vital for developing therapies for cardiovascular, oncological, and autoimmune diseases.

The RAAS represents a multifunctional signaling network whose complexity extends far beyond classical cardiovascular regulation. The dynamic balance between the harmful ACE/Ang II/AT1R axis and the protective ACE2/ Ang-(1-7)/Mas axis, together with the counter-regulatory role of AT2R, determines physiological and pathological outcomes across multiple organ systems. Understanding subcellular mechanisms—from mitochondrial activation to nuclear regulation and from caveolar signaling to post-translational modifications—provides the rational foundation for innovative therapeutic strategies.

Further research is key to uncovering therapeutic targets and prevention strategies to improve patient management and clinical outcomes.

## Abbreviations

The following abbreviations are used in this manuscript:

RAAS	renin–angiotensin–aldosterone system
ACE	angiotensin-converting enzyme system
ANG	Angiotensinogen
AKT	Protein kinase B
APA	Aminopeptidase A
ATR	Angiotensin receptor
ADH	Antidiuretic hormone
MASR	MAS receptor
ERK	extracellular signal-regulated kinase
PI3K	phosphoinositide 3-kinase
NO	Nitric oxide
COX-2	cyclo-oxygenase-2
FOXO1	forkhead box O1
ROS	reactive oxygen species
VSMC	vascular smooth muscle cell
MrgD	Mas-related G protein-coupled receptor D
HDAC	histone deacetylase
sGC	soluble guanylate cyclase
MAPK	Mitogen-activated protein kinases
SHP-1	src homology region 2 domain-containing phosphatase-1
NICD	intracellular Notch domain
AAA	aorto-abdominal
TAA	thoracic aneurysm
iNOS	inducible oxide nitric sinthase, inducible nitric oxide synthase
eNOS	endothelial nitric oxide synthase
